# Molecular subtype concordance and metastatic patterns in muscle‐invasive bladder cancer

**DOI:** 10.1002/2056-4538.70088

**Published:** 2026-03-27

**Authors:** Csilla Olah, Dániel Juhász, Melinda Váradi, Henning Reis, Mulham Al‐Nader, Osama Mahmoud, Barbara T. Grünwald, Viktor Grünwald, Boris Hadaschik, Péter Nyirády, Tibor Szarvas

**Affiliations:** ^1^ Department of Urology University of Duisburg‐Essen Essen Germany; ^2^ Department of Urology Semmelweis University Budapest Hungary; ^3^ Institute of Pathology University Medicine Essen, University of Duisburg‐Essen Essen Germany; ^4^ Department of Urology, Qena Faculty of Medicine South Valley University Qena Egypt; ^5^ Carolus Institute for Urologic Oncology University of Duisburg‐Essen Essen Germany

**Keywords:** muscle‐invasive bladder cancer, molecular subtypes, multiorgan involvement, survival outcome, Lund Taxonomy

## Abstract

Molecular subtypes are potential prognostic and predictive tools in muscle‐invasive bladder cancer (MIBC). However, subtype concordance between primary tumors and metastases, as well as subtype‐specific differences in metastatic patterns, remain poorly characterized. The present study aimed to evaluate the concordance of molecular subtypes between primary tumors and matched lymph node (LN) metastases and to explore their association with metastatic patterns. Gene expression–based molecular subtypes were determined according to the five‐tiered Lund Taxonomy in 182 primary tumor samples and 34 matched LN metastases from patients with MIBC who underwent upfront radical cystectomy. Subtypes identified in the primary tumors were compared with those in matched positive LNs and patterns of distant metastasis were analyzed. In addition, the association between molecular and histological subtypes was also investigated. We found an overall 62% subtype concordance between primary tumors and corresponding LN metastases, with complete concordance in the basal/squamous subtype, lower concordance in the luminal subtypes (genomically unstable: 67%; urothelial‐like: 57%), and low concordance (33%) in the mesenchymal‐like (Mes) subtype. Luminal subtypes were associated with LN‐only metastases and less frequent distant metastases. In contrast, the Mes subtype was associated with a higher rate of distant metastases (43%), and more frequent multiorgan involvement (≥3 organs: 40%). Higher expression of the mesenchymal gene *CDH2* and the neuronal‐differentiation genes *GNG4* and *ENO2* was associated with a higher number of metastatic sites. Gene expression–based molecular subtypes may change between primary MIBCs and matched LN metastases, and these differences appear to be subtype‐dependent. Mes subtype and the expression of *CDH2* as well as *GNG4* and *ENO2* are associated with more frequent and extensive metastases, indicating highly aggressive forms of MIBC.

## Introduction

Bladder cancer (BC) is a clinically heterogeneous disease with an estimated global incidence of 614,298 new cases per year [1]. Approximately 25% of cases are muscle‐invasive bladder cancer (MIBC) and subsequent lymph node (LN) or distant metastases develop in approximately half of these patients, most commonly within the first 3 years after radical cystectomy (RC). Overall survival (OS) for metastatic MIBC patients treated with platinum‐based chemotherapy (CTx) ranges from 9 to 26 months [2, 3]. The therapeutic landscape for metastatic BC patients has recently expanded with the availability of immune checkpoint inhibitors (ICIs), Nectin‐4– and Her2‐targeting antibody‐drug conjugates (ADCs), and the pan‐FGFR inhibitor erdafitinib, offering new opportunities to supplement or even replace long‐standing CTx‐based regimens [4, 5, 6, 7, 8]. Despite these therapeutic advances, metastatic BC continues to be associated with high mortality [9].

MIBC is a biologically diverse disease with variable pathology, molecular profiles, and clinical outcomes. Gene expression (GE) patterns define distinct molecular subtypes with specific prognosis and therapy sensitivity [10, 11]. Several studies have examined the association between molecular subtypes and CTx efficacy across various treatment settings. Early findings suggested that patients with the basal/squamous (Ba/Sq) subtype benefit in terms of OS from neoadjuvant CTx [12]. In contrast, other studies reported better pathological response rates and OS in luminal tumors – urothelial‐like (Uro) and genomically unstable (GU) subtypes [11]. Similarly, the Uro subtype showed improved OS in adjuvant and metastatic CTx‐treated cohorts [13, 14, 15]. However, comparisons across studies remain challenging due to different molecular classification systems [Consensus, Lund Taxonomy (LundTax), TCGA, GSC], variable clinical endpoints, and the limited comparability between GE‐ and immunohistochemistry‐based subtypes. The LundTax is the only classifying system defining five molecular subtypes at both GE and protein levels, enabling direct transcriptomic–proteomic comparison. Few studies have explored molecular subtypes in relation to ICI response, suggesting higher sensitivity in small‐cell/neuroendocrine‐like (Sc/Ne‐like) and GU subtypes [16, 17, 18], while molecular subtypes have not yet been evaluated in patients treated with ADCs. Notably, the prospective GUSTO trial is currently assessing TCGA‐subtype‐guided versus standard neoadjuvant CTx [19].

Since systemic treatments mainly target metastatic lesions, the molecular subtypes of distant metastases are of clinical importance. However, the molecular characterization of metastatic sites has received considerably less attention, and even fewer studies have directly compared the molecular subtypes of primary tumors with those of corresponding LN or distant metastases, primarily due to the limited availability of paired samples [15, 20, 21]. Thus, there is limited information regarding the stability of molecular subtypes in LN or distant metastases; however, such knowledge may have important implications for understanding subtype‐specific therapeutic sensitivities.

Therefore, in the present work, we determined GE‐based molecular subtypes in the primary tumor and matched positive LNs of MIBC patients to assess potential molecular subtype shifts in LN metastases. In addition, we evaluated the association between molecular subtypes of primary tumors and their metastatic patterns, and correlated molecular subtypes with histological subtypes, providing insights into disease progression and thereby predicting tumor aggressiveness.

## Materials and methods

### Patient cohort

The present study included 182 patients with urothelial MIBC, who underwent RC at the Department of Urology, University of Duisburg‐Essen, between 2003 and 2018. Inclusion criteria were a pathological stage of ≥pT2 at RC and a tumor cell content of at least 50% in the available tumor specimen. Histological subtypes of the tumors were identified by an expert uro‐pathologist (HR) [22]. A sub‐cohort of patients (*n* = 75) received adjuvant CTx within 90 days of RC, whereas another subcohort (*n* = 16) received palliative CTx following subsequent progression. In 34 cases, matched LN metastases were also available for analysis. OS was used as the primary endpoint and was calculated as the time from RC to death or last follow‐up. The study was approved by the institutional ethics commission and conducted in accordance with the Declaration of Helsinki (15‐6400‐BO). The cohort partly overlapped with a previously published dataset (*n* = 159) that was analyzed for the predictive value of molecular subtypes for adjuvant CTx, including patients with and without treatment [14].

### Molecular subtype analysis

Molecular subtypes were determined based on the GE profiles of primary tumors and matched positive LNs according to the LundTax classification using a 48‐gene panel and a classifier rule set as described earlier. The following molecular subtypes were determined: urothelial‐like (Uro), GU, Ba/Sq, mesenchymal‐like (Mes), Sc/Ne‐like [14, 23]. All samples were identified as urothelial MIBC. In each case, the FFPE tissue block with the highest tumor content was selected and the total tumor area was marked on the corresponding H&E‐stained tissue sections for RNA isolation. The presence or absence of specific histological subtypes did not influence block selection or the marking of the region of interest. RNA was extracted from unstained FFPE sections of RC and lymphadenectomy specimens using the RNeasy DSP FFPE Kit (Qiagen, Hilden, Germany). GE was measured using the NanoString nCounter Analysis System (NanoString Technologies, Seattle, WA, USA), and data were analyzed with the nSolver software (v.4.0). GE levels were normalized to two reference genes (*GAPDH* and *TBP*), six internal positive controls, and eight internal negative controls.

### Statistical analysis

The chi‐square test or Fisher's exact test (for small sample sizes, *n* ≤ 5) was used to compare the distribution of molecular and histological subtypes between metastatic and nonmetastatic MIBC patients (LN0M0 versus LN + M0 and versus M+), as well as between different distant metastatic sites. The Mann–Whitney test was applied to compare GE levels and identify differentially expressed genes between patient subcohorts with distinct metastatic sites. Cox univariable analysis was performed among patients with distant metastases to evaluate OS differences across patient subcohorts with different metastatic sites. Kaplan–Meier curves were used to visualize OS differences between patients with LN‐only and distant metastases within each molecular subtype group and between patients with one, two or multiorgan metastases. Sankey plots were generated in R (version 4.2.1, Vienna, Austria) to illustrate molecular subtype heterogeneity between samples with positive LNs and/or distant metastases. GE patterns were visualized on heatmaps (Morpheus, https://software.broadinstitute.org/morpheus). Statistical analyses were conducted using IBM SPSS Statistics for Windows, version 25 (IBM Corp., Armonk, NY, USA). All statistical tests were two‐sided, and *p* ≤ 0.05 was considered statistically significant.

## Results

### Patients' characteristics

This study included 182 urothelial MIBC patients who were treated with RC (Table 1). LN positivity and distant metastases were identified in 88 (48%) and 14 (8%) patients, respectively, at the time of RC by the pathologist, while a further 31 (17%) patients experienced disease progression after surgery – 7 (4%) with LN‐only metastases, 15 (8%) with distant metastasis, and 9 (5%) with both LN‐and distant metastases – detected by imaging. The most common sites of distant metastasis were the lungs (*n* = 21, 12%), liver (*n* = 14, 8%), and bone (*n* = 11, 6%). Based on tumor stage or disease progression, 91 (50%) patients received CTx regimens. The median OS for the whole cohort was 12.6 months. Molecular subtypes according to the LundTax were determined for all primary tumors and for matched LNs (*n* = 34) (Table 2). The cohort included urothelial tumors; however, histologic subtypes in addition to pure NOS (urothelial carcinoma, not otherwise specified) were identified in 84 (46%) samples.

**Table 1:** Patient characteristics

Clinical data	*n* (%)
Total number of patients	182
Age at baseline median (range)	68.2 (39.3–90.3)
Sex	
Male	133 (73)
Female	49 (27)
Stage	
pT2	21 (12)
pT3	104 (57)
pT4	51 (28)
na	6 (3)
Metastases at RC	
LN+	88 (48)
M+	14 (8)
Metastatic progression after RC	
LN+	16 (9)
M+	24 (13)
Localization of all distant metastases	
Lung	21 (12)
Liver	14 (8)
Bone	11 (6)
Other	12 (7)
Chemotherapy	91 (50)
Gem/Cis	78 (43)
Carboplatin	2 (1)
Cis/MTX	3 (2)
MVAC	8 (4)
No chemotherapy	91 (50)
Number of patients died	134 (74)
Follow‐up time in months, median (range)	12.6 (0.1–162.7)

Cis/MTX, cisplatin/methotrexate; Gem/Cis, gemcitabine/cisplatin; LN, lymph node; M, distant metastasis; MVAC, methotrexate, vinblastine, doxorubicin, and cisplatin; na, not available; RC, radical cystectomy.

**Table 2:** Molecular subtype distribution in primary tumors stratified by metastatic pattern of patients (LN0M0 versus LN+ only or M+ cases) and in matched available positive LNs

Cohort	Whole cohort	LN0M0	LN+ only	M+	Whole cohort with available LN+ samples
Sample type	Primary TU, *n* (%)	Primary TU, *n* (%)	Primary TU, *n* (%)	Primary TU, *n* (%)	LN+, *n* (%)
Lund Taxonomy	*n* = 182	*n* = 69	*n* = 75	*n* = 38	*n* = 34
Urothelial‐like	66 (36)	21 (30)	37 (49)	8 (21)	10 (29)
Basal/squamous	52 (29)	27 (39)	14 (19)	11 (29)	8 (24)
Genomically unstable	33 (18)	10 (14)	16 (21)	7 (18)	8 (24)
Mesenchymal‐like	23 (13)	7 (10)	6 (8)	10 (26)	7 (21)
Small‐cell/neuroendocrine‐like	8 (4)	4 (6)	2 (3)	2 (5)	1 (3)

LN+, lymph node metastasis; M+, distant metastasis; TU, primary tumor.

### Comparison of molecular subtypes between primary tumors and LN metastases

**Figure 1:**
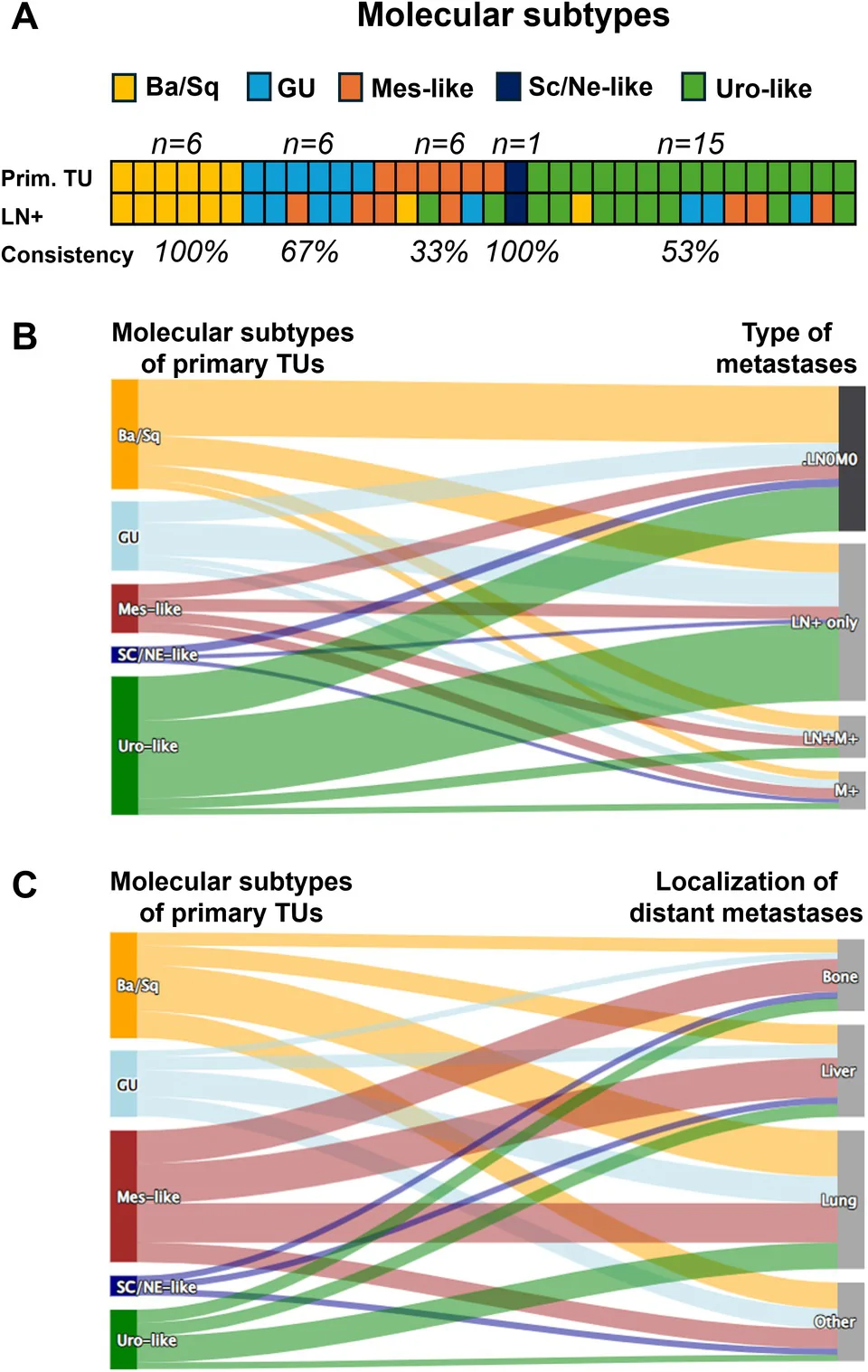
Molecular subtype heterogeneity and metastatic patterns in muscle‐invasive bladder cancer. (A) Comparison of molecular subtypes between primary tumors and matched positive lymph nodes. (B) Metastatic potential and (C) distant metastatic patterns of different molecular subtypes. Ba/Sq, basal/squamous; GU, genomically unstable; LN+, positive lymph node; M+, distant metastasis; Mes, mesenchymal‐like; Sc/Ne‐like, small‐cell/neuroendocrine‐like; Tu, tumor; Uro, urothelial‐like.

Molecular subtype concordance between primary tumors and matched positive LNs was observed in 21 cases (62%). The Ba/Sq subtype showed the highest subtype stability, with 100% concordance. In contrast, luminal subtypes (GU, Uro) demonstrated lower consistency (67% and 53%, respectively). The lowest concordance was observed in the Mes subtype, with only 33% overlap. Sc/Ne‐like is rare, occurring in less than 5% of MIBC tumors. In the one Sc/Ne‐like case with an available corresponding positive LN, the same subtype was present in the metastasis (Figure 1A).

### Metastatic potential of different molecular subtypes in primary tumors

Comparison of metastatic potential between different GE‐based molecular subtypes revealed that the Ba/Sq tumors had a slightly lower rate (48%; 25/52) of LN or distant metastasis, followed by Sc/Ne‐like (50%; 4/8), Uro (68%; 45/66), GU (70%; 23/33), and Mes (70%; 16/23).

When considering LN‐only metastasis, Uro tumors showed the highest rate (56%, 37/66), which was significantly higher compared with Ba/Sq tumors (27%, 14/52; *p* = 0.012) and Sc/Ne‐like tumors (25%, 2/8; *p* = 0.036) (Figure 1B; supplementary material, Figure S1A).

When considering only distant metastases, most of the subtypes showed similar frequencies of metastatic spread (Uro: 8/66; 12%, GU: 7/33; 21%, Ba/Sq: 11/52; 21%, Sc/Ne‐like: 2/8; 25%), while the Mes subtype exhibited the highest distant metastatic potential (10/23), which was significantly higher compared with the Uro subtype (8/66) (43% versus 12%, *p* = 0.001) (Figure 1B, Table 2).

### Metastatic patterns of different molecular subtypes

Overall, 58 distant metastases were found in 38 of the 182 (21%) patients. The most frequent sites of distant metastasis were the lungs (12%), liver (8%), and bone (6%) for the whole cohort (Table 1); these represented 55%, 37% and 29%, respectively, of patients with distant metastasis. Except for Sc/Ne‐like, all molecular subtypes were frequently involved in lung metastases, with ≥50% of distant metastatic patients with Uro (4/8; 50%), GU (4/7; 57%), Ba/Sq (7/11; 64%), or Mes (6/10; 60%) subtypes exhibiting lung involvement. Mes subtype was overrepresented in liver metastases (6/10; 60%) compared to Uro (2/8; 25%), Ba/Sq (3/11; 27%) and GU (2/7; 29%). Similarly, the Mes subtype had more frequent bone metastasis (5/10; 50%) compared to Uro (2/8; 25%), Ba/Sq (2/11; 18%), and GU (1/7; 14%). The Sc/Ne‐like subtype was rare, with only two cases presenting distant metastases – one with bone involvement and the other with multiple organ metastases, including the liver, colon, and spleen (Figure 1C and supplementary material, Figure S1B).

A subcohort of patients with multiorgan involvement by metastasis was identified; these patients had at least three separate distant metastases across different organs. Among all patients with distant metastatic disease, 21% (8/38) exhibited multiorgan metastases. Seven patients with multiorgan metastases (88%) died within 1 year following RC. The Mes subtype was predominant in this cohort, accounting for 50% (4/8) of cases, a proportion higher than in Uro (0/8; 0%), Ba/Sq (2/11; 18%), and GU (1/7; 14%) subtypes. Among the two metastatic samples classified as Sc/Ne‐like, one exhibited multiorgan metastases (supplementary material, Figure S1B).

### Association between histological and molecular subtypes and their metastatic potential

Histological subtypes were represented in 46% (84/182) of primary tumors. The most frequent were squamous urothelial carcinoma (Sq) in 20% (37/182) and micropapillary urothelial carcinoma (MPUC) in 9% (17/182), followed by sarcomatoid carcinoma (SARC) in 4% (7/182) and small‐cell/neuroendocrine carcinoma (SC/NE) in 3% (6/182). Sq histological subtypes were mostly identified as Ba/Sq molecular subtypes (28/37; 76%). Patients with the MPUC histological subtype were predominantly classified as GU (11/17; 65%) and less frequently as Uro (4/17; 24%), both representing molecular subtypes with high luminal GE. The SARC variant was classified mostly as Mes in 63% (5/8) and Ba/Sq in 25% (2/8) of cases. SC/NE tumors were assigned to the Sc/Ne‐like molecular subtype in 33% (2/6) of cases and to the Mes subtype in 50% (3/6). Pure NOS tumors were mostly identified as Uro (51/98; 52%), followed by GU (18/98; 18%) (supplementary material, Figure S2). Similar to the Mes molecular subtype, the SARC histological subtype showed a significantly higher rate of distant metastases (6/8, 75%) compared with the pure NOS subtype (*p* < 0.001). Distant metastases were also more frequent in the SC/NE histological subtype (3/6; 50%; *p* = 0.049) (supplementary material, Figure S1C). Consistent with the molecular subtype findings, Sq and MPUC subtypes were overrepresented in lung metastases (3/6; 50% and 4/5; 80%), while SARC and SC/NE tumors were enriched in liver metastases (4/6; 67% and 2/3; 67%, respectively). In addition, SC/NE cases also showed higher multiorgan involvement (2/3; 67%) (supplementary material, Figure S1D).

### Survival analyses

Among patients with distant metastases, liver metastasis was identified as a significant risk factor for poor patient outcome (HR: 2.225, 95% CI: 1.055–4.691, *p* = 0.036), while lung and bone metastases showed no significant association with OS.

Interestingly, among patients with luminal tumors (GU and Uro), no significant OS difference was observed between LN‐only and distant metastatic cases (Uro *p* = 0.519, GU *p* = 0.965). In contrast, in the Ba/Sq and Mes subtypes, those with LN‐only metastases demonstrated significantly better OS compared with patients exhibiting distant metastases (*p* = 0.002 and *p* = 0.004, respectively) (supplementary material, Figure S3). Among patients with distant metastases, those with the Mes subtype exhibited the poorest prognosis (log‐rank: *p* = 0.141), with a median OS of only 3.7 months (range: 0.3–18.8 months). Patients with Uro, GU and Ba/Sq subtypes showed similar median OS (Uro: 8.2 months; range: 1.4–68.3 months, GU: 8.0 months, range: 0.9–98.1 months, Ba/Sq: 9.0 months, range: 2.1–22.4 months). Patients with multiorgan involvement had the shortest median OS (3.7 months; range: 2.2–12.6) among patients with metastases. However, the differences were not significant (log‐rank: *p* = 0.245) compared with patients who had two (9.0 months; range: 0.3–68.3) or one (7.4 months; range: 0.9–98.1) metastatic site (supplementary material, Figure S4).

### Single GE associated with metastatic patterns

Consistent with the high rate of LN‐only metastases in the Uro subtype, some of the luminal genes, including *CYP2J2*, *FGFR3*, *FOXA1*, *GATA3*, *PPARG*, *UPK1A*, and *UPK2*, were significantly overexpressed in LN‐only metastatic compared to distant metastatic cases (supplementary material, Figure S5).

**Figure 2:**
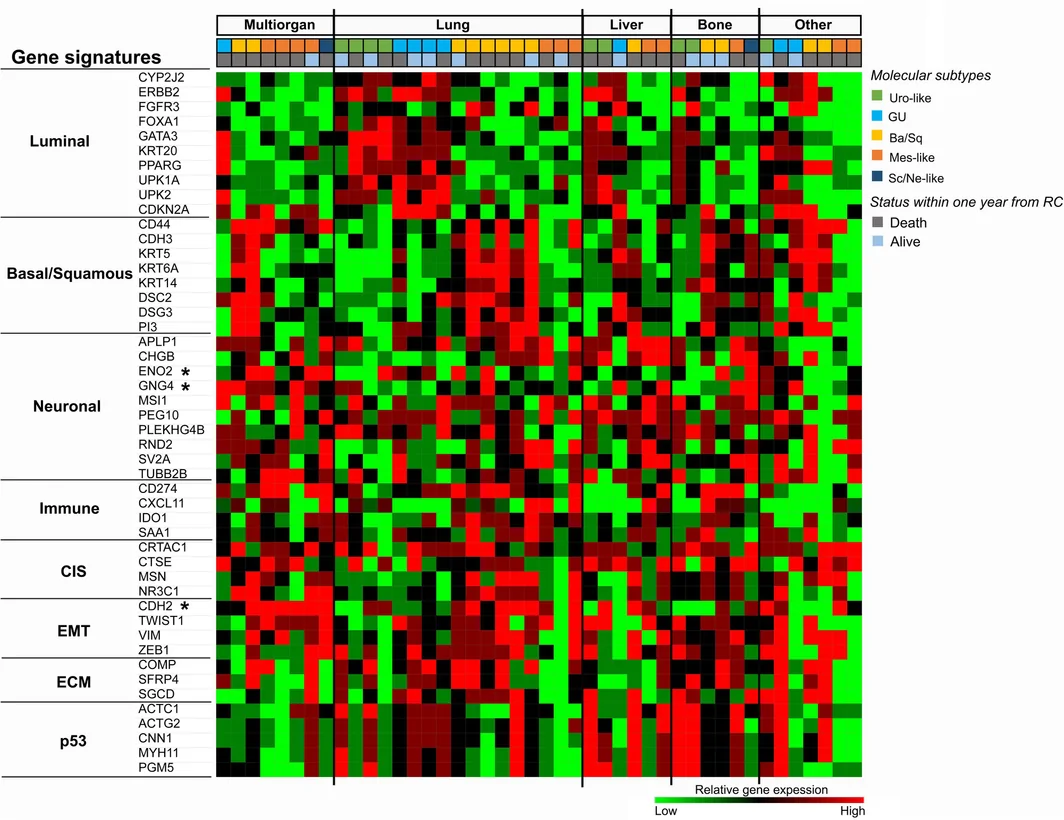
Gene expression profiles of molecular subtypes in muscle‐invasive bladder cancer, stratified by the localization of distant metastases. Ba/Sq, basal/squamous; CIS, carcinoma *in situ*; ECM, extracellular matrix; EMT, epithelial‐mesenchymal transition; GU, genomically unstable; Mes, mesenchymal‐like; RC, radical cystectomy; Sc/Ne‐like, small‐cell/neuroendocrine‐like; Uro, urothelial‐like. *Significant differences in gene expression in samples with multiorgan involvement compared to non‐multiorgan cases.

According to the finding that Mes subtype showed a higher number of distant metastases, the mesenchymal marker *CDH2* (*p* = 0.023), and in addition two neuronal‐differentiation genes: *GNG4* (*p* = 0.008) and *ENO2* (*p* = 0.032) were significantly upregulated in the primary tumor of patients who had multiorgan metastasis (Figure 2 and supplementary material, Figure S6A). Additionally, GE levels of *CDH2* (*p* = 0.036 and *p* = 0.046) and *GNG4* (*p* = 0.008 and *p* = 0.043) showed a significantly increasing trend with the number of metastatic sites (supplementary material, Figure S6B). *FOXA1* GE was significantly higher in patients with lung metastases (*p* = 0.041), whereas *GNG4* expression was upregulated in patients with liver metastases compared to those with metastases at other distant sites (*p* = 0.026) (supplementary material, Figure S6C, D).

## Discussion

In the present study, comparison of GE–based subtypes between primary tumors and matched LN metastases revealed subtype‐dependent heterogeneity, with a 62% overall concordance. Molecular subtype analysis of primary tumors revealed that Mes subtype had the highest (43%) and the Uro subtype the lowest (12%) distant metastatic potential over the follow‐up period of surgical patients. Luminal subtypes (Uro and GU) more often showed LN‐only metastases, whereas Mes tumors displayed frequent multiorgan spread and poorer prognosis.

Molecular subtypes of MIBC differ in their biology and therapeutic sensitivities, underscoring their clinical relevance. However, most studies have characterized molecular subtypes in the primary tumors, which are often already removed before systemic treatment is initiated. Consequently, in clinical practice, it is the metastatic lesions that are exposed to systemic therapy. This raises important questions regarding the concordance and stability of molecular subtypes between primary and metastatic sites, and whether profiling of metastatic tissues may better inform therapy selection. To date, only a limited number of studies have directly compared GE‐based molecular subtypes between matched primary and metastatic sites. An early study by Sjödahl *et al* analyzed 67 primary tumors and matched LNs, with molecular subtype information available for 28 pairs, and reported a concordance rate of 71% (20/28), including 60% (3/5) concordance for the Ba/Sq subtype and 88% (7/8) for the Uro subtype [20]. A subsequent study by Cox *et al* analyzed 20 primary MIBCs and matched distant metastases, reporting only 45% (9/20) subtype concordance according to the Consensus classification. The highest concordance was observed in Ba/Sq tumors (80%, 4/5), whereas luminal papillary, luminal unstable, and stroma‐rich subtypes showed lower consistency (33%; 2/6, 1/3, 2/6) [21]. Another study that analyzed nine primary tumors and matched distant metastases reported that in 67% of cases (6/9), the predominant Consensus subtype of the primary tumor was also identified in metastatic lesions. However, the high coexistence of different subtypes within the tumors made comparisons difficult [9]. Overall, available data suggest that molecular subtype concordance between primary tumors and matched LN or distant metastases ranges from 45% to 71% based on five‐ or six‐tiered GE‐based classifications. Our analysis, conducted in a slightly larger cohort of patients, revealed a 62% (21/34) concordance of molecular subtypes between primary MIBCs and matched metastatic LNs, which is comparable to previously reported observations. The Ba/Sq subtype showed complete concordance (100%), whereas luminal subtypes (Uro and GU) demonstrated lower concordance rates of 53% and 67%, respectively. The Mes subtype exhibited the lowest concordance, with only 33% agreement.

In addition to intertumoral heterogeneity, we assessed the metastatic pattern of different molecular subtypes determined from primary MIBCs. Ba/Sq and Sc/Ne‐like showed the lowest metastatic potential, whereas Mes tumors exhibited the highest incidence of distant metastases. Uro tumors were most often associated with LN‐only metastases, suggesting subtype‐specific differences in metastatic patterns that may impact disease progression. Most studies have focused on the prognostic or therapy‐predictive value of molecular subtypes determined in primary tumors, often in nonmetastatic cohorts, whereas less attention has been paid to their metastatic patterns. An early study reported that bone metastases were observed only in p53‐like subtypes (9/16, 56%), but not in luminal or basal subtypes [24]. A Lund study including 70 metastatic cases found that MIBCs with positive LNs are mostly classified as Uro and GU subtypes. Regarding distant metastases, Uro tumors predominantly spread to the lung, GU tumors were enriched in ‘other’ sites beyond the typical lung, liver, or bone, and Ba/Sq tumors were rarely associated with bone metastases [25]. An independent study identified the subtypes from metastatic lesions. The authors described that Consensus luminal papillary tumors were more frequently observed in lung metastases, Ba/Sq tumors at ‘other’ metastatic sites, and luminal unstable tumors were overrepresented in LN and liver metastases [9]. In our MIBC cohort, primary tumors with high luminal GE (Uro, GU) were also overrepresented in patients with LN‐only metastases. Lung metastases were frequent across the Uro, GU, and Ba/Sq subtypes, while liver and bone metastases were mainly seen in the Mes subtype. The Mes subtype also showed the highest metastatic potential with frequent multiorgan spread. Multiorgan involvement occurred in 40% of Mes cases, representing half of all multiorgan metastatic cases in the cohort. In contrast, none of the Uro subtype patients showed multiorgan involvement. Seven of eight patients with multiorgan metastases died within 1 year after RC, indicating very aggressive disease and questioning the indication for radical surgery.

The association between molecular and histological subtypes has been previously confirmed. High concordance was observed between Sq histology and the Ba/Sq subtype, while pure NOS tumors were associated with luminal gene and protein expression [26]. In accordance with these results, we found that Sq histology was mainly identified as Ba/Sq (76%). Pure NOS tumors were most frequently Uro (52%), followed by GU (18%). SARC tumors were predominantly identified as Mes (63%). Their metastatic patterns were similar, showing a relationship between molecular and histological subtypes. Sq was enriched in lung metastases (50%), and SARC tumors exhibited a higher rate of distant spread (75%), particularly to the liver (67%). SC/NE histology was overrepresented in cases with multiorgan involvement (67%), which is consistent with the elevated expression of two neuronal‐differentiation genes (*GNG4* and *ENO2*) in patients with a higher number of metastases.

While multiorgan spread has been reported only in case reports in the context of MIBC, it is already well described in breast cancer, where clinicopathological parameters and molecular patterns of primary tumor are associated with metastatic site distribution, the potential of multiorgan spread, and patient survival [27]. Bone is the most common metastatic site of breast cancer, and its colonization may promote subsequent multiorgan spread. In a mouse model, Zhang *et al* demonstrated that cancer cells undergo a phenotypic shift within the bone microenvironment, losing their luminal characteristics and acquiring stem cell‐like features, which enable further metastatic dissemination [28]. Similarly, in the present study, 75% of patients with multiorgan metastases (6/8) had bone involvement. In line with the high occurrence of Mes tumors among patients with multiorgan metastases, GE of *CDH2* was also elevated. *CDH2* encodes the mesenchymal protein N‐cadherin that promotes stromal invasion and metastasis through epithelial‐to‐mesenchymal transition and is associated with therapeutic resistance across multiple cancer types including BC [29]. A further study that determined the LundTax molecular subtypes, mostly from primary tumors (91%), and evaluated their predictive value for CTx response in a metastatic urothelial carcinoma cohort found that patients with the Mes subtype had a significantly lower response rate to CTx and shorter OS [30]. Accordingly, our previous study revealed that high‐risk nonmetastatic MIBC patients derived no significant benefit from adjuvant CTx [14]. These findings, together with our observation of the Mes subtype's high metastatic potential and frequent multiorgan involvement, support its characterization as an aggressive and therapy‐resistant tumor phenotype.

The Sc/Ne‐like molecular subtype is considered a rare but highly aggressive form of BC [31]. A recent study reported that this subtype is found four times more frequently in distant metastases than in primary BC samples [9]. Consistent with these findings, in the present study, two neuronal‐differentiation genes, *GNG4* and *ENO2*, showed significantly increasing GE by increasing the number of metastatic sites. On the other hand, only half of the patients developed distant metastases; however, one of the two patients with metastatic disease with the Sc/Ne‐like molecular subtype presented multiorgan metastases. Similarly, half of the patients with SC/NE histology had distant metastases, and two in three of these patients had multiorgan spread. However, the low number of cases did not allow firm conclusions about this rare subtype.

This study has several limitations, primarily related to its retrospective design and the small sample sizes within different molecular subtype groups and metastatic site subcohorts. These constraints limited our ability to adequately adjust for potential confounders and to draw definitive conclusions about the prognostic significance of the findings. Despite the limited sample size, our study cohort is the largest to date for the analysis of GE‐based molecular subtypes in paired samples of primary bladder tumor tissue and corresponding LN metastases.

In conclusion, our findings complement previous GE–based analyses, indicating potential subtype shifts between primary MIBCs and matched positive LNs, with Ba/Sq being the most stable and Uro and GU showing lower consistency. The Mes subtype shows high metastatic potential, frequently spreading to multiple distant organs, and is associated with aggressive behavior. Combined with previous results of poor chemotherapy responsiveness, these characteristics predict very poor prognosis for patients with the Mes subtype.

## Author contributions statement

CO: writing – original draft, data analysis, visualization. DJ, MV, MA‐N, OM: data analysis, methodology. HR: conceptualization, methodology, data analysis, writing – review and editing. BH and BTG: resources, supervision, writing –review and editing. VG and PN: writing – review and editing. TS: conceptualization, methodology, funding acquisition, data analysis, writing – original draft.

## Supporting information


**Figure S1.** Distribution of molecular and histological subtypes among patients with lymph node and distant metastases and among patients with different sites of distant metastases
**Figure S2.** Overlap between molecular and histological subtypes and their associations with lymph node involvement and patterns of distant metastasis, including multi‐organ involvement
**Figure S3.** Overall survival of patients with different molecular subtypes stratified by the presence of lymph node and/or distant metastases
**Figure S4.** Overall survival of patients with distant metastasis stratified by the number of metastases in different organs
**Figure S5.** Gene expression profiles of molecular subtypes in metastatic bladder cancer, stratified by the presence of lymph node‐only versus distant metastases
**Figure S6.** Expression levels of significantly differentially expressed molecular subtype–related genes

## Data Availability

Raw gene expression data and patient‐level data are available on request from the corresponding author.
