# Supplementary material for: Molecular subtype concordance and metastatic patterns in muscle‐invasive bladder cancer
Source: J Pathol Clin Res. 2026 Mar 27;12(3):e70088. doi: 10.1002/2056-4538.70088 (PMC13581105; doi:10.1002/2056-4538.70088)
Supplement: Supplementary file 1 [file CJP2-12-e70088-s001.pdf]

Molecular subtype concordance and metastatic patterns in muscle-invasive bladder cancer

C Oláh *et al*. *J Pathol Clin Res* https://doi.org/10.1002/2056-4538.70088

Supplementary Figures S1–S6

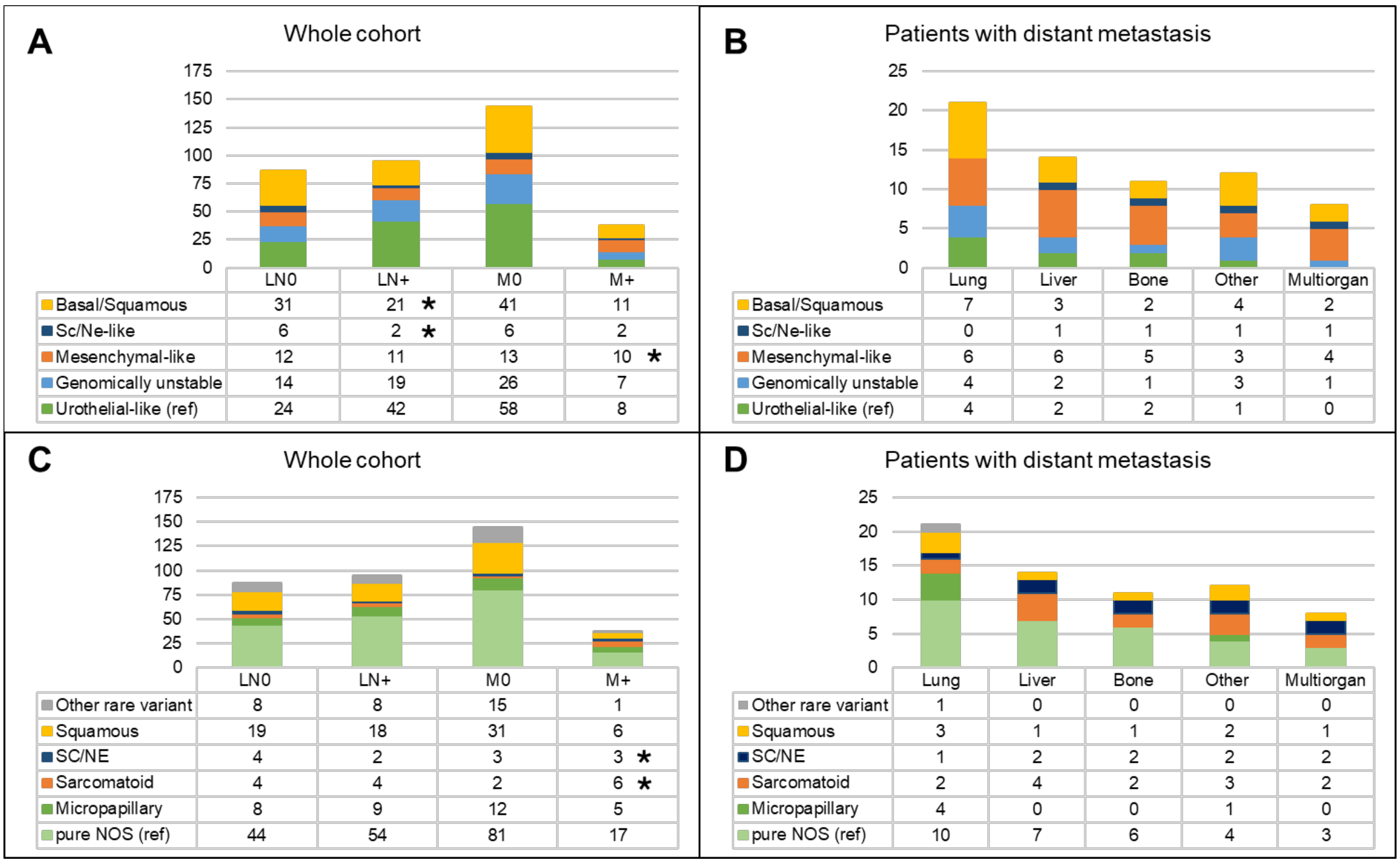

Figure S1. Distribution of (A) molecular and (C) histological subtypes among patients with lymph node (LN) and distant metastases (M+) and among patients with different sites of distant metastases (B and D). Significant differences between subtypes, tested using the Chi² test or Fisher's exact test (for expected number of patients < 5), are marked. LN+: lymph node metastasis, M+: distant metastasis, pure NOS: urothelial carcinoma, not otherwise specified, Sc/Ne-like: small-cell/neuroendocrine-like molecular subtype, SC/NE: small-cell/neuroendocrine carcinoma

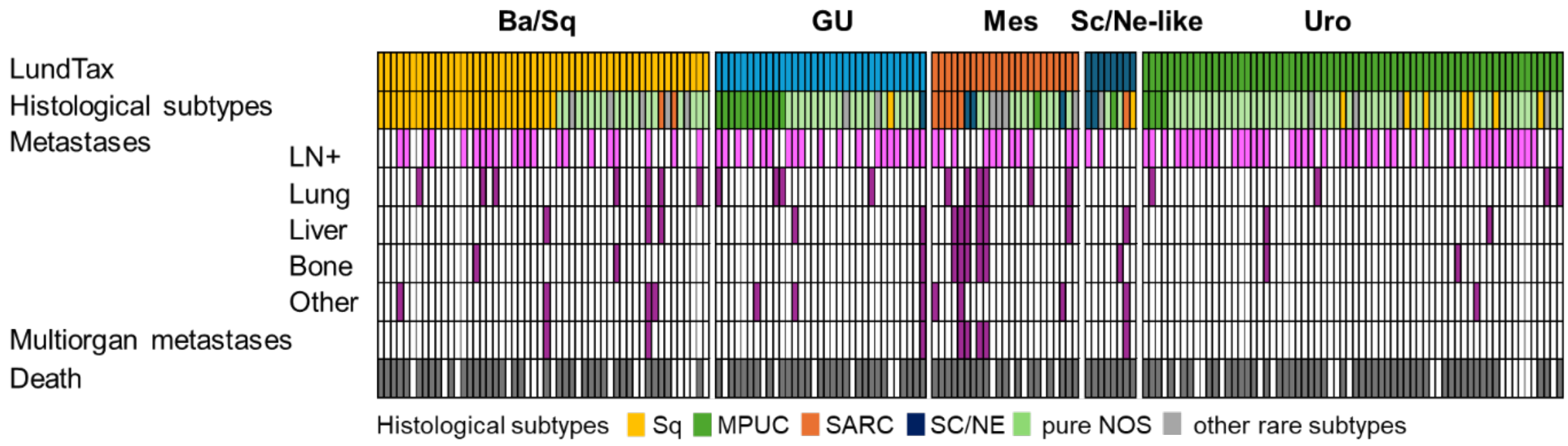

Figure S2. Overlap between molecular and histological subtypes and their associations with lymph node involvement and patterns of distant metastasis, including multi-organ involvement. LundTax: molecular subtypes according to Lund Taxonomy, LN+: lymph node metastasis, Ba/Sq: basal/squamous, GU: genomically unstable, Mes: mesenchymal-like, Sc/Ne-like: small-cell/neuroendocrine-like molecular subtype, Uro: urothelial-like, Sq: squamous histological subtype, MPUC: micropapillary urothelial carcinoma, SARC: sarcomatoid, SC/NE: small-cell/neuroendocrine carcinoma, NOS: urothelial carcinoma, not otherwise specified.

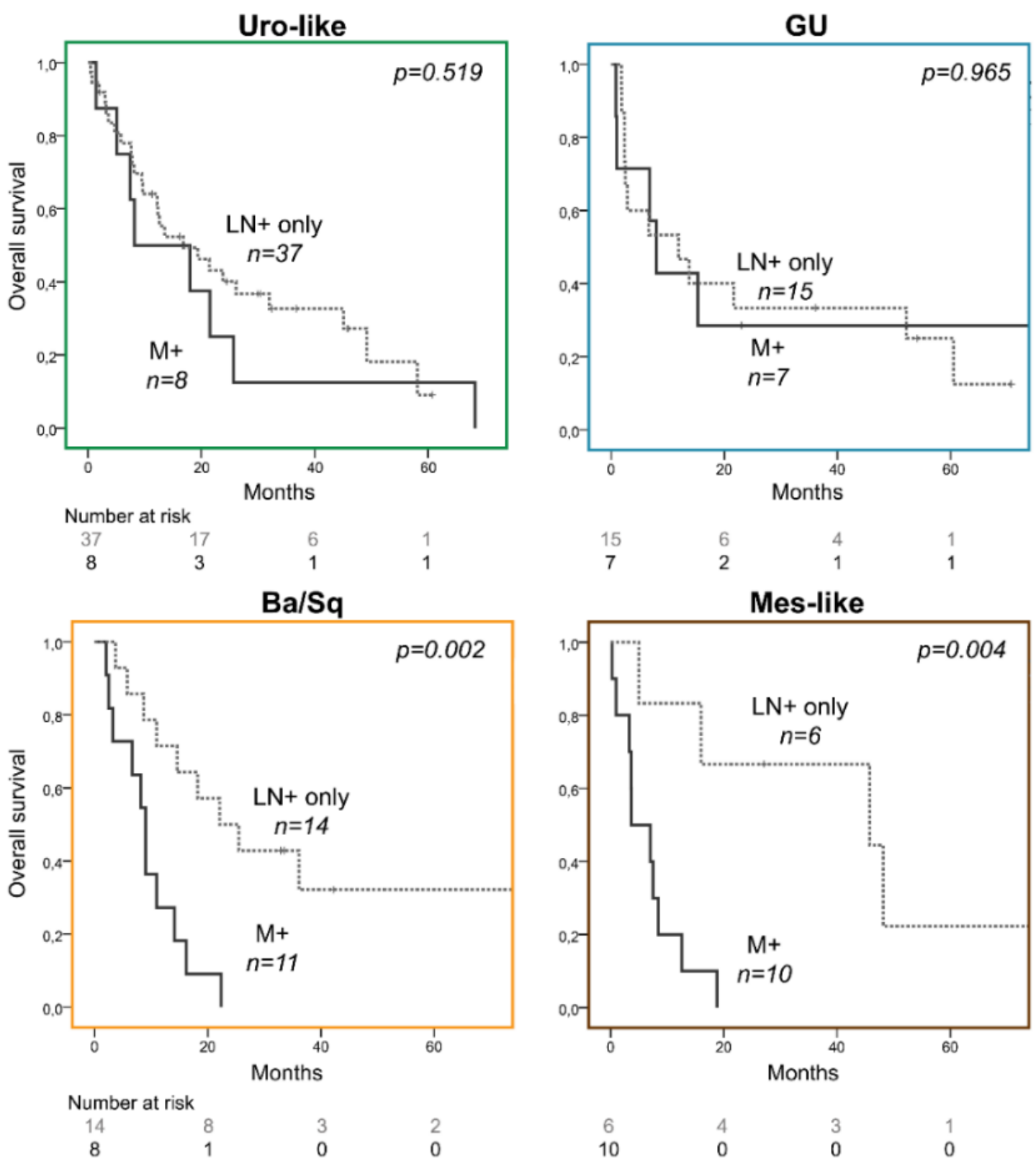

Figure S3. Overall survival of patients with different molecular subtypes stratified by the presence of lymph node and/or distant metastases. Ba/Sq: basal/squamous, GU: genomically unstable, LN+: positive lymph node, M+: distant metastasis, Mes: mesenchymal-like, Uro: urothelial-like.

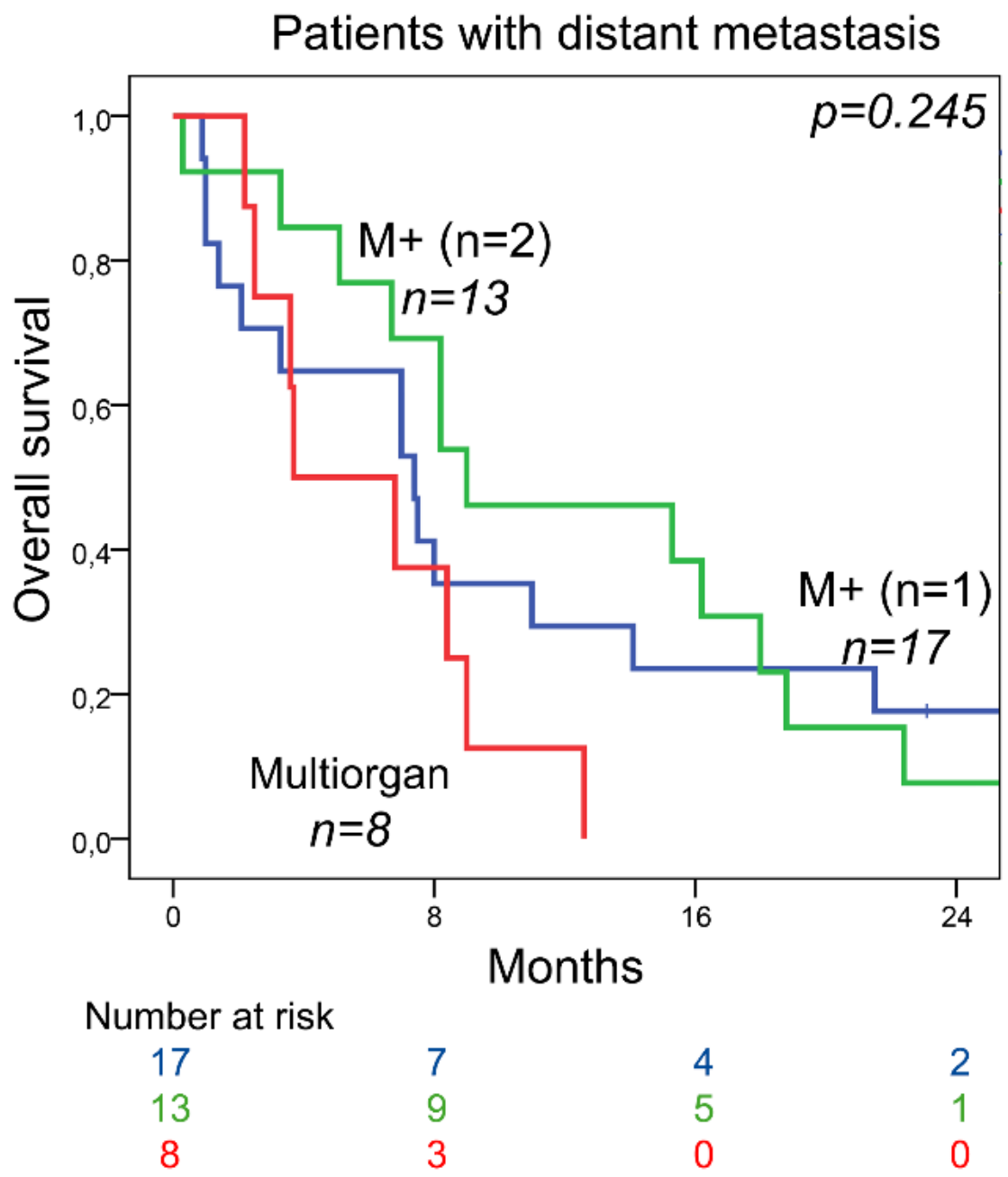

Figure S4. Overall survival of patients with distant metastasis (M+) stratified by the number of metastases in different organs (one metastatic sites versus two metastatic sites versus multiorgan metastasis).

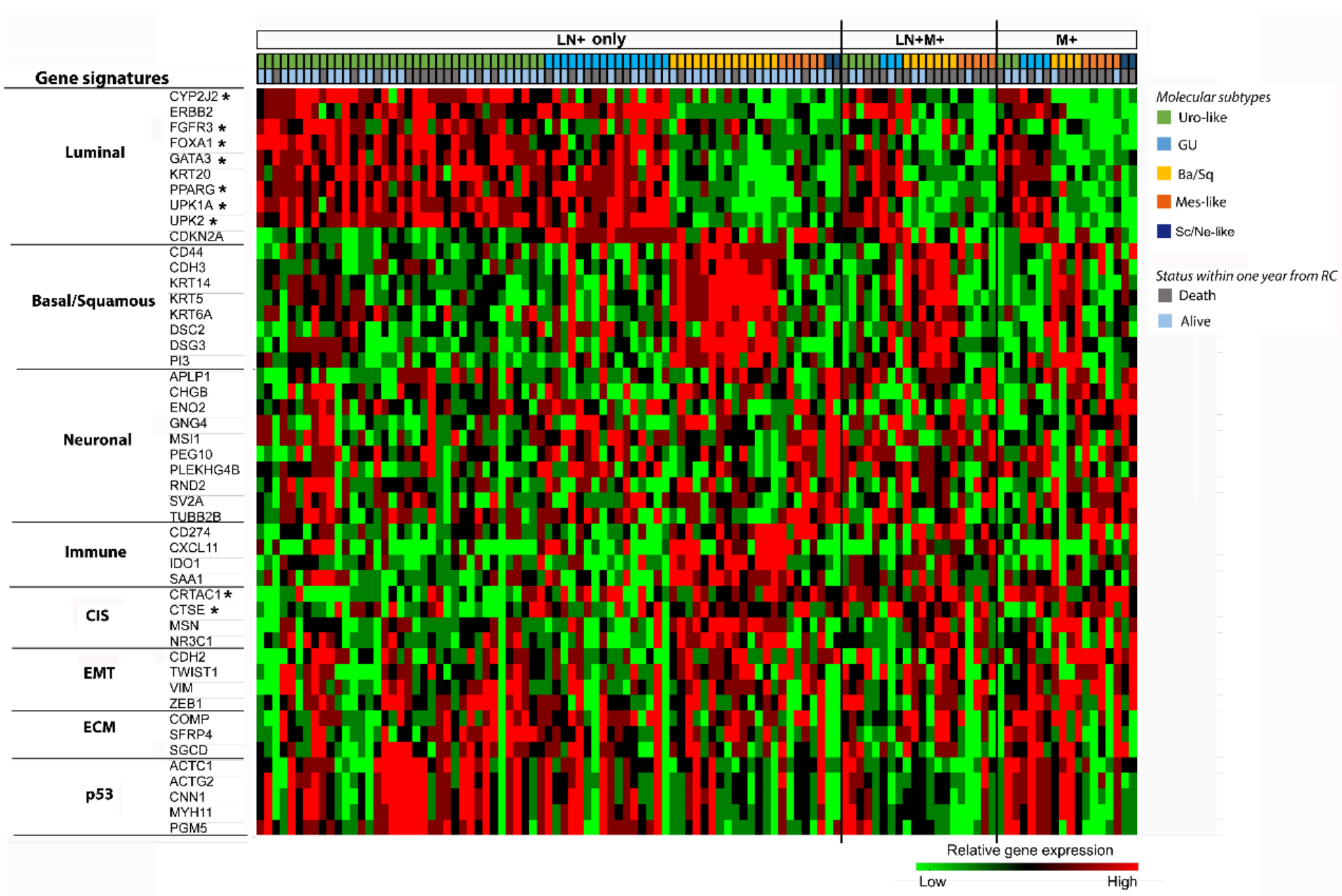

Figure S5. Gene expression profiles of molecular subtypes in metastatic bladder cancer, stratified by the presence of lymph node-only (LN+ only) versus distant metastases (M+). Ba/Sq: basal/squamous, CIS: carcinoma in situ, GU: genomically unstable, EMT: epithelial-mesenchymal transition, ECM: extracellular matrix, Mes: mesenchymal-like, RC: radical cystectomy, Sc/Ne-like: small-cell/neuroendocrine-like molecular subtype, Uro: urothelial-like. *Significant differences in gene expression in samples with multiorgan involvement compared to non-multiorgan cases.

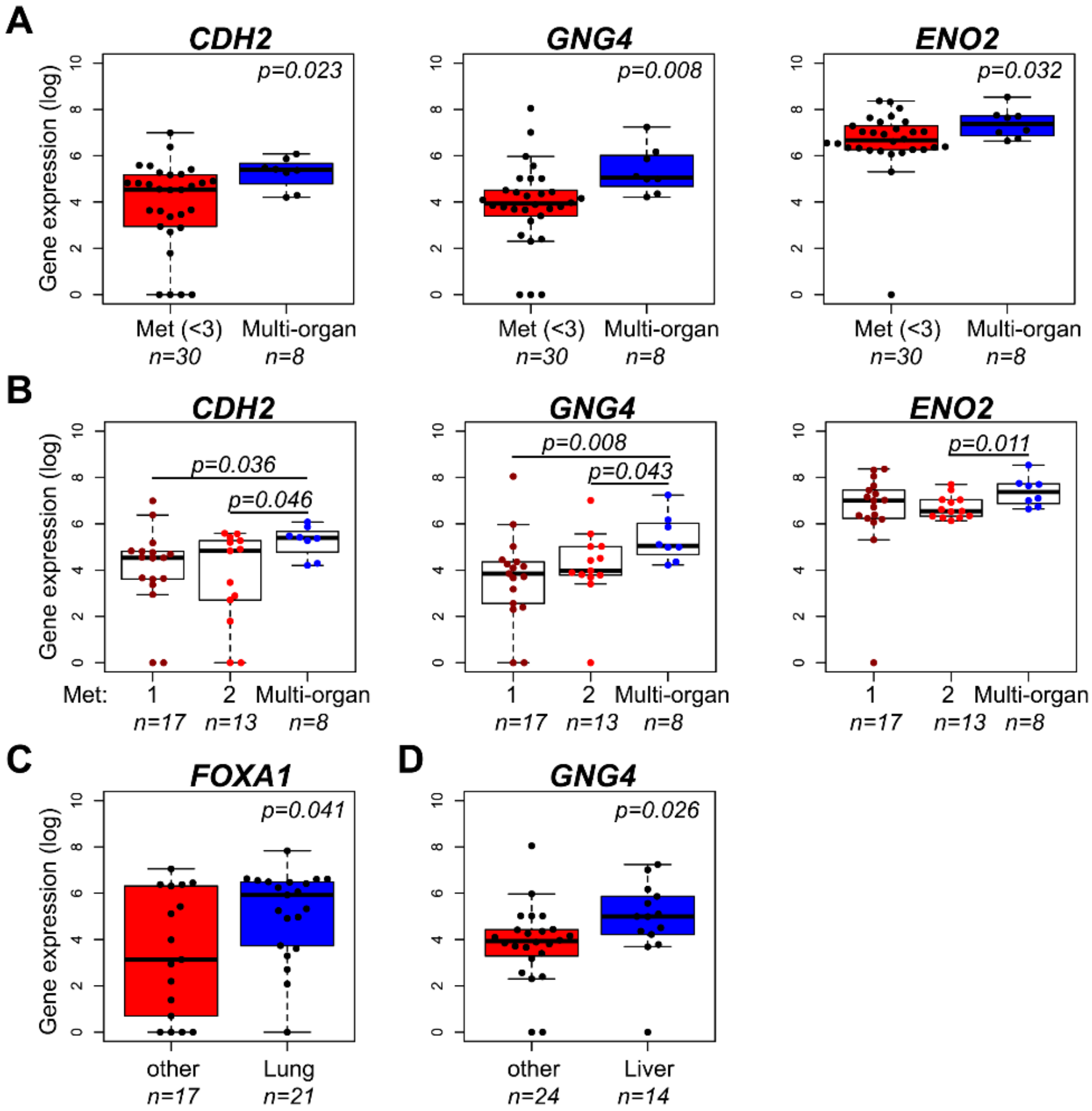

Figure S6. Expression levels of significantly differentially expressed molecular subtype–related genes in (A) patients with multiorgan versus non–multiorgan metastases, (B) patients with one, two, or multi-organ metastases, (C) lung versus other metastatic sites, and (D) liver versus other metastatic sites.
